# BRCA1/2 mutation screening in high-risk breast/ovarian cancer families and sporadic cancer patient surveilling for hidden high-risk families

**DOI:** 10.1186/1471-2350-14-61

**Published:** 2013-06-14

**Authors:** Dace Berzina, Miki Nakazawa-Miklasevica, Jekaterina Zestkova, Karina Aksenoka, Arvids Irmejs, Andris Gardovskis, Dagnija Kalniete, Janis Gardovskis, Edvins Miklasevics

**Affiliations:** 1 Institute of Oncology, Riga Stradins University, Dzirciema street 16, LV1007, Riga, Latvia

**Keywords:** *BRCA2*, Breast cancer, Hereditary cancer families, Mutation analysis

## Abstract

**Background:**

The estimated ratio of hereditary breast/ovarian cancer (HBOC) based on family history is 1.5% in Latvia. This is significantly lower than the European average of 5–10%. Molecular markers like mutations and SNPs can help distinguish HBOC patients in the sporadic breast and ovarian cancer group.

**Methods:**

50 patients diagnosed with HBOC in the Latvian Cancer Registry from January 2005 to December 2008 were screened for *BRCA1* founder mutation-negatives and subjected to targeted resequencing of *BRCA1* and *BRCA2* genes. The newly found mutations were screened for in the breast and ovarian cancer group of 1075 patients by Real Time-PCR/HRM analysis and RFLP.

**Results:**

Four *BRCA2* mutations including three novel *BRCA2* frameshift mutations and one previously known *BRCA2* frameshift mutation and one *BRCA1* splicing mutation were identified. Two of the *BRCA2* mutations were found in a group of consecutive breast cancer patients with a frequency of 0.51% and 0.38%.

**Conclusions:**

Molecular screening of sequential cancer patients is an important tool to identify HBOC families.

## Background

Breast and ovarian cancers are the most common and increasing cancers among women worldwide. From the breast/ovarian cancer cases in Europe 5–10% are diagnosed as hereditary [1] which frequently have early onset [2]. The most common approach to diagnosing hereditary cancer is to investigate family history. However, hereditary breast/ovarian cancer (HBOC) is often difficult to identify by family history due to the small size of families and uncertain family history records [3]. The incidence of hereditary cancer (diagnosed according to the National Comprehensive cancer network (NCCN) guidelines) is 1.5% of all the breast cancers in Latvia [3]. This is significantly lower than the European average. In other words, many HBOC patients may be unnoticed among the cancer patients considered sporadic, missing an opportunity to be clinically consulted for risk control. Molecular screening of all cancer patients in order to reveal pathogenic high-penetrance mutations is an obvious alternative. Mutations in the *BRCA1* and *BRCA2* genes are known as the main risk factors of HBOC and are found in about 80% of patients [4,5]. In Latvia, two founder mutations, c.4035delA and c.5266dupC, of the *BRCA1* gene dominate [6,7], but no prevalent *BRCA2* mutation has been reported as yet. Molecular screening of consecutive breast and ovarian cancer patients revealed that 3.77% of breast cancer and 9.9% of ovarian cancer patients had been harboring one of the *BRCA1* founder mutations [2]. Identification of new frequent mutations in either of these genes would promote the identification of more HBOC patients without substantial cost increases.

This study provides the results of our attempt to identify new *BRCA1/2* mutations in HBOC patients and estimate their usefulness for molecular screening to spot hidden hereditary breast/ovarian cancer patients without a significant family history.

## Methods

### Study population

50 unrelated patients who had been diagnosed with HBOC from January 2005 to December 2008, according to the NCCN guidelines.

Blood samples for the study material had been collected from consecutive 1075 breast or ovarian cancer patients at Pauls Stradins Clinical University Hospital from January 2005 to December 2008. These patients had been screened for *BRCA1* founder mutations c.181T>G (BIC: 300T>G), c.4035delA (BIC: 4154delA) and c.5266dupC (BIC: 5382insC) and found negative earlier [2]. All patients have been informed of the analyses, and they have given written consent to have their blood samples used for DNA analyses. Permission for the research project has been given by the Ethical Committee of Riga Stradins University.

Families which had at least three breast, ovarian or breast and ovarian cancer patients and one of those patients was the first degree relative to other two or the second degree relative through male were classified as HBC, HOC or HBOC families, respectively.

### *BRCA1* and *BRCA2* analysis

Genomic DNA was isolated from peripheral blood cells using the FlexiGene DNA Kit (Qiagen, Germany). Screening of the three most common *BRCA1* mutations in Latvia, c.181T>G (BIC: 300T>G), c.4035delA (BIC: 4154delA) and c.5266dupC (BIC: 5382insC), was performed by multiplex PCR. The samples without *BRCA1* founder mutations were subjected to direct sequencing of the coding regions of *BRCA1* and *BRCA2* genes and analyzed by ABI PRISM 3130 (Applied Biosystems, USA).

Screening of the *BRCA2* c.658delGT mutation was performed by Real Time PCR/High Resolution Melting (HRM) run on Rotor-Gene 6000 amplification (Qiagen, Germany). *BRCA2* c.5244delC and c.7316delG mutations were screened by restriction fragment length polymorphism (RFLP) analysis. The PCR products were digested with AluI and BccI restriction enzymes (NEB, England), respectively. All the mutations detected in HRM and RFLP were confirmed by sequencing. All the primers were as described before [8].

## Results

50 unrelated families who corresponded to the NCCN guidelines for HBOC (23 families), HBC (25) and HOC (2) were identified in Latvia from 2005 to 2008. The ethnic composition of the group matched the ethnic structure of the country: 27 Latvian (54% of the HBOC families and 59% of the residents in Latvia), 16 Russian (32% and 28%), 3 Polish (6% and 2.3%), 2 Belarusian (4% and 3.6%) and 2 Ukrainian (4% and 2.5%) families. Screening for three *BRCA1* mutations (c.181T>G, c.4035delA and c.5266dupC) revealed that 15 families were (10 HBOC, 3 HBC and 2 HOC) harboring either mutation c.4035delA (8 families) or c.5266dupC (7 families). The distribution of the *BRCA1* mutations by ethnicity was as follows: 8 Latvian (29.6% of hereditary cancer families from the same ethnic group), 1 (6%) Russian, 3 (100%) Polish, 2 (100%) Ukrainian and 1 (50%) Belarusian. From the remaining 35 patients, 30 agreed to targeted resequencing of the *BRCA1/2* genes.

Four clinically significant mutation and thirteen polymorphisms [9-15] in the *BRCA1* and *BRCA2* genes were identified by targeted resequencing (Table 1).

**Table 1:** Polymorphisms found in the BRCA1 and BRCA2 genes by exon resequencing

**Gene**	**Nucleotide change**	**Effect on protein**	**NCBI SNP**	**Clinical significance**^**1**^	**Case n=30**
BRCA1	c.2311T>C	L771L	rs16940	No	3
	c.3113A>G	E1038G	rs16941	No	2
	c.4308T>C	S1436S	rs1060915	No	4
	c.4675+1G>A	INV15+1	rs80358044	Yes	1
	c.4837A>G	S1613G	rs1799966	No	1
BRCA2	c.-41A>G	5'UTL	-	Unkown	1
	c.-26G>A	5'UTL	rs1799943	No	20
	c.658delGT	V220 (223stop)	rs80359604	Yes	1
	c.1114A>C	N372H	rs144848	No	1
	c.3396A>G	K1132K	rs1801406	No	10
	c.3807T>C	V1269V	rs543304	No	2
	c.4258G>T	D1420Y	rs28897727	No	2
	c.4563A>G	L1521L	rs206075	No	2
	c.5244delC	S1748 (1748stop)	-	Yes	1
	c.5744C>T	T1915M	rs4987117	minor	2
	c.7242A>G	S2414S	rs1799955	No	4
	c.7316delG	G2439 (2468stop)	-	Yes	1

^1^ As clinically significant were considered nonsense, frameshift and splice site mutations, as well as missense ones which are considered as such in Breast Cancer Information Core database [16].

777 consecutive breast cancer and 298 consecutive ovarian cancer patients were screened for the presence of any of the three *BRCA2* mutations found in hereditary cancer families. To detect the c.658delGT variant, Real Time PCR/HRM of exon 8 was performed. Two different melting patterns compared to the wild-type were found in 7 cases. The PCR fragments with different melting curves were sequenced and harbored either the c.658delGT or c.646delG mutation (Table 2). None of these mutations were found among the 298 ovarian cancer patients. No other carriers of mutations c.5244delC and c.7316delG were identified (Table 2).

**Table 2:** Mutations found in the BRCA2 gene in consecutive breast or ovarian cancer patients

**Nucleotide change**	**Effect on protein**	**Case (n=1075)**	**Diagnosis/age**
c.646delG	A216 (229stop)	3	BC/39,44,58
c.658delGT	V220 (223stop)	4	BC/43,51,55,73
c.5244delC	S1748 (1748stop)	0	
c.7316delG	G2439 (2468stop)	0	

**Figure 1:**
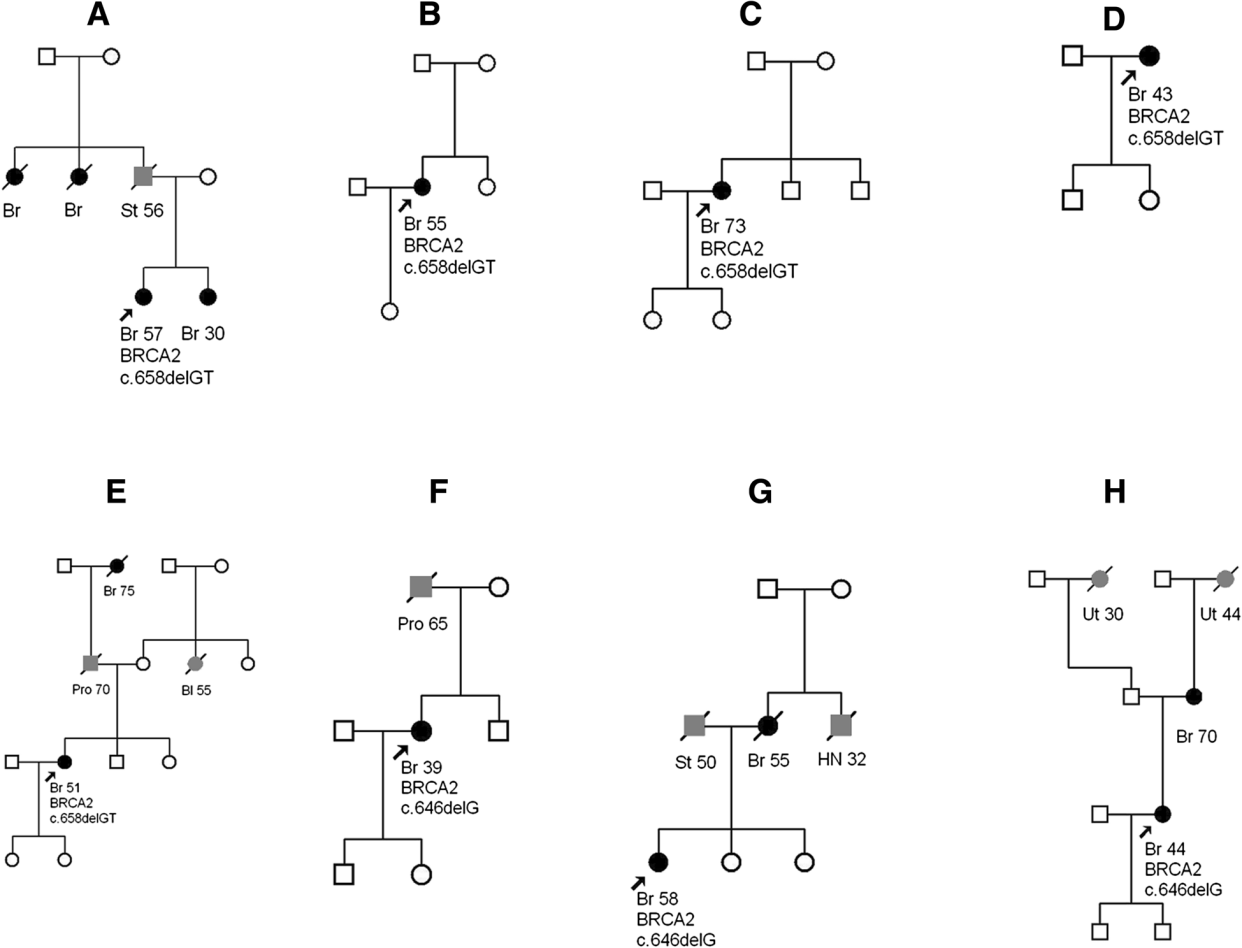
**Family pedigrees of the patients with an identified *BRCA2 *c.658delGT (A-E) and c.646delG (F-H) mutation.** A family pedigree of patient from HBOC group, **A** – **H** – patients from consecutive patients group. Breast cancer patients are marked in black. Patients with other cancer localization are marked in gray (Pro, prostate; Ut, uterus; Bl, bladder; HN, head and neck; St, stomach). The patients in whom *BRCA2* mutations were found by molecular screening are indicated by arrows and information about mutation added to proband.

One HBOC patient and four patients from sporadic cancer group were identified as carriers of the c.658delGT mutation. In three non-HBOC patients the c.646delG mutation was found. Figure 1 shows family pedigrees of patients. Size of family for all patients is relatively small and some patients don’t have previously known cancer cases in family. None of pedigrees of patients from consecutive patients group corresponded to criteria of HBOC.

## Discussion and conclusions

In a previous study, we had screened for *BRCA1* founder mutations c.4034delA and c.5266dupC in consecutive breast/ovarian cancer patients, and it showed that 57.5% of mutation carriers did not correspond to the clinical criteria of HBC or HBOC [2]. This indicates that a significant number of patients carrying *BRCA1* or *BRCA2* mutation are still missing an opportunity of proper counseling or surveillance of other family members. The main reason for an insufficient detection of HBOC patients based on family histories is due to the small family size resulting into a small number of relatives. In families with a larger number of relatives it is easier to diagnose hereditary cancer. The significant difference in the size of the families who were diagnosed with hereditary cancer syndromes, according to defined criteria, and in the families with non-diagnostic findings has been described previously in the population screening of the Valka region in Latvia [3]. The mean number of blood relatives within the families with hereditary cancer syndromes, according to criteria, was 13.6, whereas it was 9.5 for the families not diagnosed with hereditary cancer syndrome but whose members were carriers of the *BRCA1* founder mutation. In the case of hereditary breast cancer, clinical findings based on family history do not overlap with the results of molecular screening, and molecular screening reveals more mutation carriers than clinical criteria.

Our findings show that in total seven patients not diagnosed with HBOC based on family history were harboring deleterious mutation in *BRCA2*. One of the mutations which was found in the non-HBOC group of this study, c.658delGT, is listed in the BIC database (886delGT) [16]. This mutation has also been reported as a genetic risk factor of brain tumor development in the Fanconi anaemia group D1 [13,17]. The frequency of the mutation c.658delGT in *BRCA2* is 0.9% in Polish ovarian-stomach and ovarian cancer families [18], 1.9% in Portuguese breast cancer families [19] and 0.09% in American breast cancer patients [20]. The frequency of the c.658delGT mutation in *BRCA2* in this study was 2% in the HBOC patient group and 0.51% in the consecutive breast cancer patient group. This is the most common *BRCA2* mutation in Latvia. To our best knowledge, the *BRCA2* c.646delG mutation has not been reported as yet.

Despite finding 10 *BRCA2* mutation carriers in the breast cancer patients, we did not find any *BRCA2* mutation carriers in the ovarian cancer patients. Due to the small number of ovarian cancer patients in the HBOC patient group, we might have missed the *BRCA2* mutations which tend to affect the risk of ovarian cancer. Inspecting the pedigree charts of *BRCA2* mutation carriers for ovarian cancer families, just one ovarian cancer family member in a *BRCA2* c.7316delG carrier family was found. However, the relation between ovarian cancer and the *BRCA2* mutation is uncertain because we did not analyze the mutation status of other family members except the proband.

In this study, we found *BRCA2* mutations with probable founder effect in patients without a significant family history using molecular screening. It can be useful to screen all consecutive breast cancer patients for the specific *BRCA1* and *BRCA2* mutations with founder effect.

## Abbreviations

HBOC: Hereditary breast/ovarian cancer; HBC: Hereditary breast cancer; HOC: Hereditary ovarian cancer; NCCN: National comprehensive cancer network; Real Time-PCR/HRM: Real time polymerase chain reaction/high resolution melting; RFLP: Restriction fragment length polymorphism.

## Competing interests

The authors declare that they have no conflict of interests.

## Authors’ contributions

DB: drafting the article, analysis and interpretation of data; MNM: drafting the article, analysis and interpretation of data; JK: analysis and interpretation of data; KA: analysis and interpretation of data; AI: revising of article for critically important intellectual content; AG: analysis and interpretation of data; DK: analysis and interpretation of data; JG: revising of article for critically important intellectual content; EM: design of experiments, final approval of the version to be published. All authors have read and approved the final manuscript.

## Pre-publication history

The pre-publication history for this paper can be accessed here:

http://www.biomedcentral.com/1471-2350/14/61/prepub
